# Trajectory tracking of changes digital divide prediction factors in the elderly through machine learning

**DOI:** 10.1371/journal.pone.0281291

**Published:** 2023-02-10

**Authors:** Jung Ryeol Park, Yituo Feng

**Affiliations:** 1 Technology Policy Research Division, Electronics and Telecommunications Research Institute (ETRI), Daejeon, South Korea; 2 Management Information Systems, Chungbuk National University, Cheongju, South Korea; Hanyang University, REPUBLIC OF KOREA

## Abstract

**Research motivation:**

Recently, the digital divide problem among elderly individuals has been intensifying. A larger problem is that the level of use of digital technology varies from person to person. Therefore, a digital divide may even exist among elderly individuals. Considering the recent accelerating digital transformation in our society, it is highly likely that elderly individuals are experiencing many difficulties in their daily life. Therefore, it is necessary to quickly address and manage these difficulties.

**Research objective:**

This study aims to predict the digital divide in the elderly population and provide essential insights into managing it. To this end, predictive analysis is performed using public data and machine learning techniques.

**Methods and materials:**

This study used data from the ‘2020 Report on Digital Information Divide Survey’ published by the Korea National Information Society Agency. In establishing the prediction model, various independent variables were used. Ten variables with high importance for predicting the digital divide were identified and used as critical, independent variables to increase the convenience of analyzing the model. The data were divided into 70% for training and 30% for testing. The model was trained on the training set, and the model’s predictive accuracy was analyzed on the test set. The prediction accuracy was analyzed using logistic regression (LR), support vector machine (SVM), K-nearest neighbor (KNN), decision tree (DT), and eXtreme gradient boosting (XGBoost). A convolutional neural network (CNN) was used to further improve the accuracy. In addition, the importance of variables was analyzed using data from 2019 before the COVID-19 outbreak, and the results were compared with the results from 2020.

**Results:**

The study results showed that the variables with high importance in the 2020 data predicting the digital divide of elderly individuals were the demographic perspective, internet usage perspective, self-efficacy perspective, and social connectedness perspective. These variables, as well as the social support perspective, were highly important in 2019. The highest prediction accuracy was achieved using the CNN-based model (accuracy: 80.4%), followed by the XGBoost model (accuracy: 79%) and LR model (accuracy: 78.3%). The lowest accuracy (accuracy: 72.6%) was obtained using the DT model.

**Discussion:**

The results of this analysis suggest that support that can strengthen the practical connection of elderly individuals through digital devices is becoming more critical than ever in a situation where digital transformation is accelerating in various fields. In addition, it is necessary to comprehensively use classification algorithms from various academic fields when constructing a classification model to obtain higher prediction accuracy.

**Conclusion:**

The academic significance of this study is that the CNN, which is often employed in image and video processing, was extended and applied to a social science field using structured data to improve the accuracy of the prediction model. The practical significance of this study is that the prediction models and the analytical methodologies proposed in this article can be applied to classify elderly people affected by the digital divide, and the trained models can be used to predict the people of younger generations who may be affected by the digital divide. Another practical significance of this study is that, as a method for managing individuals who are affected by a digital divide, the self-efficacy perspective about acquiring and using ICTs and the socially connected perspective are suggested in addition to the demographic perspective and the internet usage perspective.

## Introduction

Information communication technologies (ICTs), especially the internet and the web, have changed every aspect of human life. These aspects range from individual social life to health outcomes and from the modernization of industry to the economic growth of nations [1, 2]. Owing to ICTs, many people have become capable of efficiently communicating with others, even in a noncontact environment, and easily acquiring various types of information. Despite the prospect of ICTs for enhancing the everyday lives of people, the inaccessibility of ICTs has resulted in a significant gap between those who can access, use, and benefit from these interventions and those who cannot [3–5]. This gap is now emerging as a new type of inequality in the era of ICTs [6–8]. In particular, as elderly individuals fail to keep pace with the development of ICTs and become alienated in various daily living activities, including economic, social and cultural activities, a digital divide between the older and younger generations of society emerges.

The specific discussion about the digital divide is known to have begun in 1995 when the National Telecommunications and Information Administration (NTIA) first mentioned the ‘digital divide’ in its report. Since then, this term has been defined in various ways. [9] defined the digital divide as the inequality that enhances the economic and social gaps between those who have access to new ITs and those who do not have such access. [10] defined the digital divide as the gap between individuals, households, businesses and geographic areas with regard to both those who can access or who use ICTs and those who cannot access or use ICTs. Similar to these examples, the early discussion on the digital divide was focused on physical access to ICTs [8]. Since then, physical access has increased due to the development of internet speed and the spread of ICTs, but the digital divide among individuals still remains. Therefore, arguments have been made that improving physical access to ICTs may not resolve the digital divide [6, 11], and the causes of the digital divide have been extended from physical access to multidimensional causes [12].

Most previous studies on the digital divide have focused on identifying its causes by using regression analysis or structural equation modeling. [13] argued that the factors of the digital divide are technological access, autonomy, social support, skill and types of uses. [14] reported that the accumulation of capital depends on the purpose and method of using digital information, which are considered factors in the digital divide. [15] showed that access to the internet and interest in the usage of information are higher in elderly individuals who have a higher education level and a higher income level, so a digital divide can occur even among older people. [16] conducted a study with older people and reported that the major factors of the digital divide included age, educational level, and the recognition of the need to be closer to family members. [17] argued that the major factors in the digital divide are demographic variables, including socioeconomic status, education and age. [18] showed that despite the availability of abundant information through the internet, a digital divide can occur among individuals depending on their purpose and method of using the internet and their smart devices. [19] conducted a study with older people and identified their education, income, interests in technology, computer usage before their retirement and social support as the major factors in the digital divide. [20] reported that age, education, income and digital device experience may be the major factors in the digital divide.

As described above, previous studies on the digital divide suggested that demographic and socioeconomic characteristics are the major factors [21]. One notable point is that older people are frequently mentioned as one of the social classes that experience a digital divide. According to the ‘2020 Report on Digital Information Divide Survey’ by the National Information Society Agency, the digitalization level (access, capability and usage) of older people is 68.6% and that of the age group of 70 years or higher is 38.8%, which is the lowest among the information vulnerable classes (disabled people, lower-income people, farmers, fishermen, women, and marriage immigrants). As Korea is expected to be a ‘superaged society’ in 2026 [22], the digital divide of older people is becoming more serious. In particular, as ICTs have penetrated many parts of society since the outbreak of the COVID-19 pandemic, being incapable of using ICTs not only causes inequality but also threatens one’s survival. Previous studies may have considered older people as composing a social group that undergoes a digital divide due to their limitations in vision, decreased cognitive ability and decreased social relationships [23]. This viewpoint is supported by the fact that studies have been conducted to investigate the status and seriousness of the digital divide of older people [24] to identify the relationship between the digital divide and life satisfaction [25, 26] and to explore the factors of the digital divide [19, 27]. In addition, a digital divide can occur even among older people [28, 29] because the digitalization level of elderly individuals can differ depending on their educational level, access to the internet and smart devices and interest in ICTs [15].

According to [30], people over the age of 70 in Korea have difficulty accessing digital devices, such as personal computers and smartphones. According to the authors’ survey, the smartphone possession rate of elderly individuals aged 70 years or older in Korea is 44.9%, which is much lower than that of the whole Korean population (92.3%). The low access rate of elderly people to digital devices represents the severe alienation of the elderly group over the age of 70 years. In addition, the digitalization level of those over the age of 70 years is 38.8%, which is considerably lower than that of 60–69-year-olds (78.8%), who are also considered elderly [30]. Individuals in their 60s now have consumed various media as adults since 2010, when the ICT era was advocated and Web 2.0 emerged. Therefore, this generation is often considered ‘young seniors’ rather than ‘elderly people’ because many in this generation are still active members of the community. The smartphone possession rate of those in their 60s is as high as 89.7%, which is close to that of the whole Korean population (92.3%) [30]. The percentage of 60–69-year-olds who enjoy online shopping is also very high. According to [31], the amount of online shopping by those in their 60 s increased by 171% from 2014 to 2019. Fifty to fifty-nine-year-old baby boomers, who will soon be referred to as the ‘silver generation,’ have a smartphone possession rate of 98.8% [30], which is the highest among older people. Individuals in their 50s are ranked third among all the generations who use YouTube, and the number of silver creators is continuously increasing [31], thus indicating that this generation has a high digitalization level. Therefore, the generations comprising those over the age of 50 include both the generation that has difficult access to digital devices, such as computers and smartphones, and the generation that can use these devices on command.

Considering the recent noncontact environment, elderly individuals who are undergoing a digital divide are more likely to experience difficulties in daily living. Therefore, it is necessary to screen and assist these individuals. However, most of the previous studies on the digital divide of older people have focused on the factors of the digital divide, and few studies have been conducted on how well the identified factors predict the actual occurrence of the digital divide of older people and how to assist these individuals. Studies on how to address the digital divide of elderly individuals are necessary, considering that this issue has become more serious since COVID-19 and that it is more difficult for older people to benefit from online services due to their low digitalization level [32]. Although the digital divide can occur even among older people, previous studies have focused only on the digital divide between the general population and elderly individuals and on identifying the factors related to the digital divide. However, since the noncontact environment is applied to daily living worldwide, more studies need to be conducted on how to rapidly screen those who are experiencing the digital divide and how to predict those who may undergo the digital divide.

This study was conducted to provide a method for predicting the digital divide of older people by establishing digital divide models based on public data and machine learning and performing relevant analyses. To improve prediction accuracy while following the traditional research methodologies of previous studies, this study was conducted by using logistic regression analysis as the classification method for the prediction model, XGBoost, which is a boosting method evolved from the decision tree, and CNN, which is an artificial neural network. This study presents the variables significant in predicting the people who will experience the digital divide and the considerations needed to address these variables. The highlights of this paper are as follows.

- This study confirms the existence of a digital divide, even among elderly individuals, and proposes a method for making predictions through machine learning techniques.
- Important variables in predicting the digital divide within the elderly population are the demographic perspective, internet usage perspective, self-efficacy perspective, and social connection perspective.
- CNNs, which are widely used in fields such as images and videos, have been confirmed to be effective in predicting tabular data, such as that used in this study.

## Theoretical framework

### Regression models/structural equations

This study focuses on predicting the digital divide. This approach has been explored by several authors over the past decade using two types of methodologies: regression models/structural equations and classification methods. Both methods are used to help identify influencing factors of the digital divide and determine the most relevant variables. However, in terms of prediction accuracy, classification techniques are more accurate. For instance [33], distinguished three dimensions from which to analyze internet use: quantity, variety, and type (information search, socialization, entertainment, commerce, mass media, school and work, and adult content). Through standardized regression coefficients, they concluded that age and education have a significant influence on the variety and quantity of use, noting that the most significant users are young users who have the highest level of education.

On the other hand [34], identified inequalities in the digital competencies of young elementary students. Students responded to self-assessed surveys incorporating items on cultural capital, language integration, their agreement with a focus on participatory learning, and the academic results obtained in the semester prior to the interview. Through a model of structural equations, it was demonstrated that the digital competencies of young students are conditioned by their home environment, the integration of language and cultural capital, together with the conditioning of the family and the academic marks as the most determining factors. Of particular interest is the analysis of digital competencies through their categorization, which makes it possible to establish more specific guidelines. In this context [35], distinguished four digital skills according to their purpose: operational capabilities (derived from concepts that indicate a set of basic internet use capabilities), formal capabilities (related to the correct use of the internet and the management of different connections between web resources: search engines, images, pages, links), information capabilities (related to the development of an information search strategy), and strategic capabilities (use of the internet according to specific objectives, achieving a general improvement of life). Using two linear regressions [35], concluded that the differences corresponding to the first digital divide (internet connection, computer availability) were derived from the inequalities in digital skills that correspond to the second digital divide and that both are firmly related to the user’s level of education. Age is essential for understanding the variation in operational and formal capacities, although it is not relevant for information and strategic capacities.

In a later study [36], delved into the second digital divide by studying internet uses and their differences according to socioeconomic profiles. Internet activities were grouped into seven representative activities: information, news, personal development, social interaction, leisure, commercial transactions, and online games. Through a multiple linear regression, they concluded that respondents with low educational levels use the internet in their free time for extended periods and mainly for information activities, online games, and social interaction. Unemployed people are more likely to use the internet for games and social interaction than employed people. At the same time, students are more likely to search for information and seek personal development, social interaction, and leisure than employed people. Similar results were obtained based on one’s living environment: people who live in urban areas use the internet more for social interaction than those who live in rural areas.

In multiple linear regression, there is more than one independent variable. Multiple linear regression is the expansion of a simple linear regression studying straight line mathematics with Y = β0 + β1X, where β0 is the intercept and β1 is the slope. This statistical method has been widely used because of its simple algorithm and mathematical calculation [37–39]. Previous studies have shown its reliable predictive power in applications, but the estimated regression coefficients can be significantly affected if high correlations between predictors exist as a multicollinearity issue [40]. Apart from simple linear regression, a hierarchical linear model has commonly been used to deal with more complex data with a nested nature [41]. Meanwhile, stepwise multiple regression, including the combination of the forward and backward selection techniques, has been widely adopted for its high efficiency using the minimum number of essential predictors to build a successful prediction model. However, numerous studies have pointed out the potential flaws using stepwise regression, such as multicollinearity, overfitting, and the selection of nuisance variables rather than useful variables [42, 43]. Since only numerical variables are allowed for building predictive models in multiple linear regressions, categorical predictors, including nominal and ordinal variables, must be converted to binary codes using dummy variables before modeling.

## Use of machine learning

Contrary to multiple linear regression, machine learning methods in artificial intelligence (AI) are increasingly used for prediction-related research [38, 44–50]. Machine learning is one approach to implement artificial intelligence, where computers discover new rules and patterns or make predictions about new data through data learning using algorithms [51]. Machine learning approaches can be mainly divided into supervised learning and unsupervised learning. Supervised learning is a method of labeling data and predicting future results. Supervised learning approaches can be divided into regression and classification tasks according to the characteristics of the results. In unsupervised learning, hidden patterns or structures in data are found by providing data without labels. Unsupervised learning approaches include clustering and principal components analysis. In this study, classification techniques were used in supervised learning to classify given data based on discrete expectations for the digital divide. Using the classification technique, a model to distinguish data was built using different data dimensions according to specific criteria to predict discrete results for new data [52].

The advantage of machine learning is the ability to use both categorical and numerical predictors to generate models by assessing linear and nonlinear relationships between variables and the importance of each predictor. In regression analysis, which is a traditional statistical method, when many variables are used simultaneously, the basic assumptions about independent variables, such as exogeneity and homoscedasticity, are difficult to maintain, and the high correlation between variables can cause multicollinearity [53]. In contrast, for the analysis of the accuracy of the machine learning-based prediction model, it is assumed that dependent and independent variables are associated with each other. In addition, the roles that dependent variables play in predicting independent variables are analyzed, so the predictive power is unaffected even when multicollinearity occurs [54]. Therefore, an analysis can be performed even in the presence of many variables. These machine learning classification algorithms include logistic regression (LR), support vector machine (SVM), K-nearest neighbor (KNN), decision tree (DT) ensembles, and eXtreme gradient boosting (XGBoost), convolutional neural network (CNN) [55].

The LR is an analysis technique to prove the causal relationship between the independent variable and the dependent variable. Here, the form of the dependent variable is categorical data; when there are two categories, the variable is classified as a dichotomous variable, and when there are more than three categories, the variable is classified as a polynomial variable. Categorical variables are used to solve various classification problems. The formula is as follows.


$$In\left(\frac{\pi }{1-\pi }\right)=\beta 0+\beta 1X1+\beta 2X2+\cdots +\beta pXp$$ (1)

The SVM is a method of separating data by finding optimal boundaries in three-dimensional space [56]. These boundaries are referred to as hyperplane boundaries, and given a set of data belonging to either category, the SVM is used to determine the category of the new data. The SVM is used to solve classification problems, such as pattern recognition. The formula is as follows.


f(χ)=ωTχ+b (2)

The KNN is an algorithm that looks at the closest k data around given data and determines the group to which the data belong. The distance is measured in terms of the Euclidean distance. Compared to other classifiers, the KNN is simple and easy to implement, and the training speed is fast. The formula is as follows.


f(A,B)=∣x1-x2∣+∣y1-y2∣ (3)

The DT is a commonly used data mining method for establishing classification systems based on multiple covariates or for developing prediction algorithms for a dependent variable. The inference rule is similar to a tree shape, so the decision-making process can be visually and clearly determined, and the DT is used to solve various classification problems.

XGBoost, a model developed by improving the boosting method of a decision tree, has an internal function to regularize overfitting, and internal cross-validation is performed for each trial [57]. Due to its excellent classification performance, XGBoost is often used in competitions such as Kaggle. Above all, the greatest advantage of XGBoost is its high practical usefulness. XGBoost allows for the derivation of important indices, which indicate relatively more important variables among various independent variables, so that the relative predictive power of various independent variables can be reviewed. Therefore, XGBoost was used in this study. The formula is as follows.


$$L^{\left(t\right)}=\sum_{i=1}^{n}ı(Yi,{\hat{Y}i}^{\left(t-1\right)}+f_{t}(x_{i}))+\Omega (f_{t})$$ (4)

CNNs have emerged as a solution to the problems (learning time, network size, and number of variables) of conventional multilayered neural networks (MNNs). Despite the simple structure of MNNs, MNN-based machine learning requires many data and an excessively long learning time; these problems can be addressed by using CNNs. Concerning previous studies [58], conducted a study on sales forecasting by using tabular data, such as e-commerce transaction history, and the highest prediction accuracy among all classifiers used in the study (ARIMA, FE+GBRT, DNN, and CNN) was achieved using the CNN. [59] researched stock price prediction by using tabular data, such as stock trading volume, closing price, and market price, and the highest prediction accuracy among all classifiers used in the study (MLP, RNN, LSTM, and CNN) was achieved using the CNN. [60] conducted a study on predicting treatment behavior in patients by using tabular data, such as patient information, and the highest prediction accuracy among all classifiers used in the study (ANN, LR, SVM, DT, RFT, CNN) was achieved using the CNN.

These machine learning techniques have been used in studies for credit card fraud detection, student satisfaction prediction, cyberbullying detection model construction, and youth suicide risk prediction. [61] developed a predictive model for credit card fraud detection using public data on credit card transaction records and machine learning algorithms (LR, naïve Bayes, KNN). The analysis results proved that the highest prediction accuracy was achieved using the KNN algorithm. [62] developed a youth suicide risk prediction model using public data from the Korean adolescent risk behavior survey and machine learning algorithms (LR, RF, SVM, ANN, XGBoost). The highest prediction accuracy was achieved using XGBoost. In the study of [63], a cyberbullying detection model was developed using Twitter data and machine learning algorithms (Naïve Bayes, KNN, DT, RF, SVM), and the highest prediction accuracy was achieved using the SVM. [64] developed a student satisfaction prediction model using traditional regression analysis and machine learning algorithms (KNN, SVM, Light GBM, RF, ENet), and the highest prediction accuracy was achieved using ENet. Recently [65], highlighted the frequent use of machine learning techniques for data mining, including LR, SVM, DT, and ANN. Therefore, using machine learning algorithms to solve the digital divide can be a future exploratory direction.

Machine learning techniques have gradually been used in digital divide research. Among relevant studies [66], compared the results of logistic regression and classification tree techniques to analyze the individual level of ICT adoption (computers, internet, and mobile phones), determining that ICT adoption was mainly influenced by income, computer and internet skills, and age. The authors preferred the classification trees technique because it outperformed logistic regression with a more parsimonious model. Specifically, approximately half as many variables were included in the classification tree model as in the logistic regression model. Therefore, the classification tree model is recommended for classifying an individual as an ICT adopter or nonadopter. Similarly [67], used a technique derived from the C4.5 algorithm to describe similarities and differences among a series of municipality classes that present different percentages of internet presence in households. For the internet presence analysis, some classification rules describing cities’ digital divide profiles were generated. Additionally [68], used public data from the Digital Skill Survey of the Spanish National Institute of Statistics and a decision tree to establish a model for predicting the digital divide and performed relevant analyses. The authors identified educational level, age, occupation and household income as the major factors in the digital divide and reported that retirees and homemakers are affected by the digital divide.

Most previous studies employed only traditional logistic regression analysis or decision trees. However, as various classification algorithms have emerged through the development of artificial intelligence (AI), other classification methods can be added to traditional methods to increase the accuracy of prediction models. Therefore, in this study, we attempted to achieve higher prediction accuracy by using SVM, KNN, XGBoost, and CNN, as well as LR and DT, which were used in previous studies.

Additionally, given the different structures and natures of datasets, including the number of variables, dimensionality, and cardinality of predictors, that can substantially influence the accuracy of each algorithm, there is no best machine learning or statistical method for prediction accuracy [38, 44, 47–50, 69]. Although previous studies have shown machine learning algorithms to outperform multiple linear regressions, especially in handling complicated models or datasets with high complexity, most machine learning methods are considered black boxes and are uninterpretable [70–72]. Consequently, there is a controversial tradeoff between prediction accuracy and a model’s interpretability for making decisions using simple and transparent models such as multiple linear regression or potentially more accurate but complicated black box machine learning models. In social sciences, prediction is important, but interpreting the results is also critical. Therefore, to increase the convenience of model analysis while using many independent variables, this study was performed by extracting the variables that are highly important in predicting the ‘digitalization level’. For variable extraction, XGBoost’s variable importance calculation algorithm was used (more details are introduced in the methodology below). The results of this study can be used as basic data for policy establishment to solve the digital divide as a social problem.

## Materials and methods

**Fig 1:**
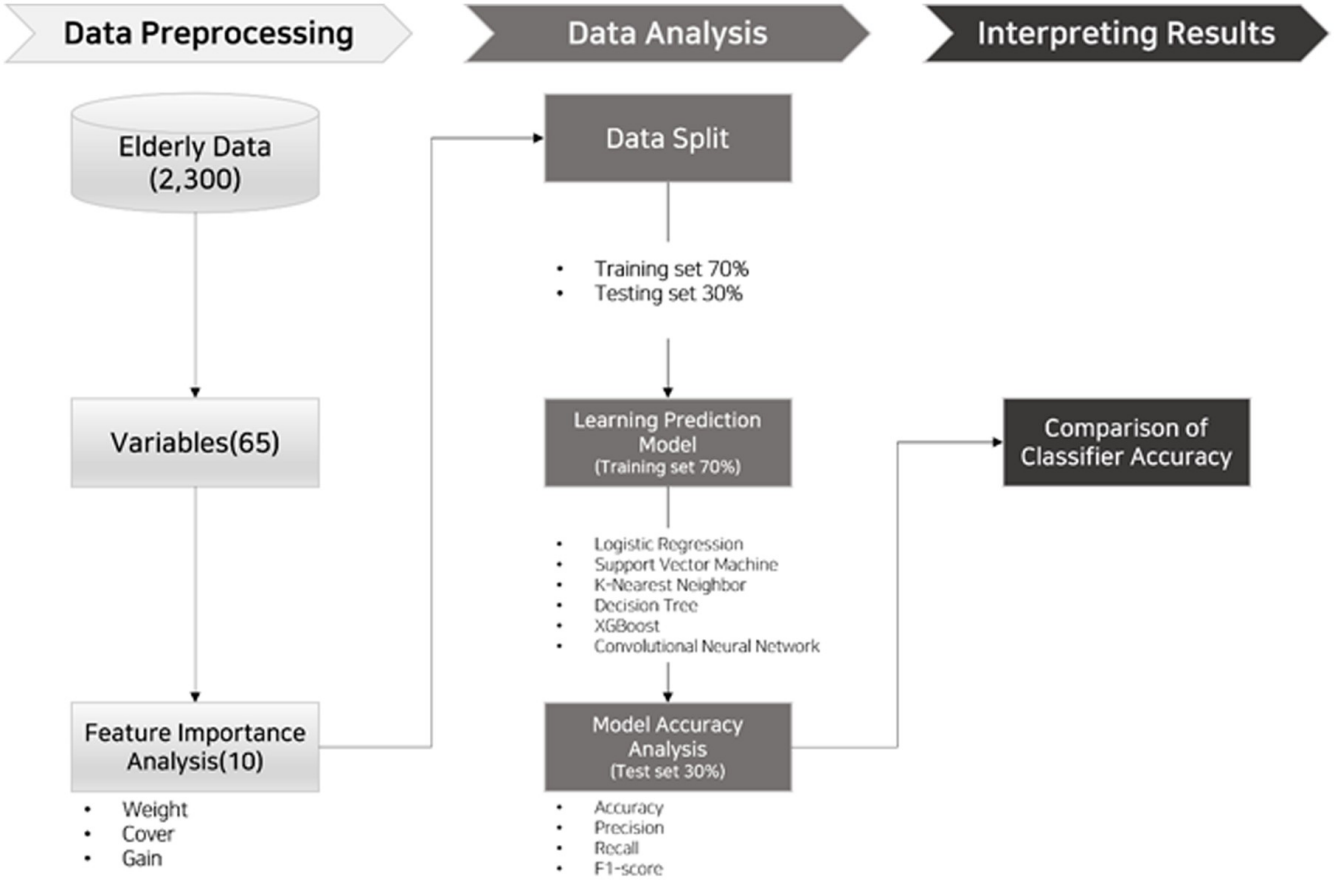
Research process.

This study aims to predict the digital divide of the older population. Therefore, this study was conducted in three stages: data preprocessing, data analysis, and interpretation of the results, as shown in Fig 1 below.

### Data

The purpose of this study was to predict the digital divide of elderly individuals. The data from the ‘2020 Report on Digital Information Divide Survey’ by the National Information Society Agency were used in this study. The survey was conducted as a face-to-face survey with 15,000 subjects in Korea for 3 months from September to December 2022. The questionnaire, shown in Table 1, included questions about the level of access to digital information, the level of digitalization capability, the level of digital information utilization, internet usage, the attitude toward using digital devices, and the change in internet usage due to COVID-19. The data included 2,300 samples, and the demographic characteristics are shown in Table 2. We also collected 2019 data for comparison between post coronavirus and precoronavirus. The 2019 data included a total of 15,000 samples and 2,300 elderly people.

**Table 1:** Measurement items.

Sortation	Category	Measurement Items
Digital informatization level	Digital information access level	Do you have wired or wireless information devices?
		Do you have internet access available at all times?
	Digital information capability level	PC literacy
		Ability to use mobile digital devices
	Level of digital information utilization	Are you using wired or mobile internet?
		Do you use a variety of internet services? (news search, community activities, product purchases, etc.)
		High level of internet use (information production, sharing, social participation, etc.)
Information usage attitude and others	Related to internet nonuse	Reasons for not using the internet
		Intention to use the internet in the future
	Digital device usage attitudes	Motivation for using digital devices
		Attitude toward using digital devices
		Digital device utilization performance
		Satisfaction with daily life categories
		Overall satisfaction with life
	Related to COVID-19	Changes in internet usage due to COVID-19
		Changes in service usage due to COVID-19
		Recognition of internet/mobile services related to COVID-19
		COVID-19–related internet/mobile experience
		Changes in life caused by COVID-19

※ Related to COVID-19 is only for 2020 data.

**Table 2:** Demographic characteristics.

Sortation	Category	Number of Samples	
		2019	2020
Sex	Male	1,065	1,068
	Female	1,235	1,232
Education	Less than elementary school graduate	457	137
	Middle school graduate	612	298
	High school graduate	1,018	1,519
	University graduate or higher	212	346
Occupation	Agriculture/Forestry/Fishing	127	80
	Service/Sales	747	539
	Production	323	492
	Professional management/Office worker	96	135
	Housewife	686	742
	Jobless/Other	321	312
Income	Less than 1 million won	228	80
	1 million won to 1.99 million won	519	258
	2 million won to 2.99 million won	507	437
	3 million won to 3.99 million won	416	529
	Over 4 million won	630	996
Total		2,300	2,300

### Dependent variable

**Fig 2:**
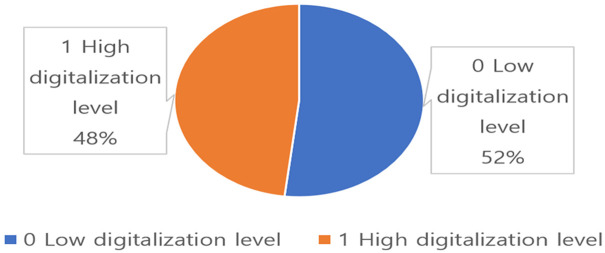
Digitalization level.

In this study, a binary dependent variable, whose values are 0 and 1, was prepared to predict the digital divide within the elderly population. Eighteen questions were used to measure the level of access to digital information, the level of digitalization capability and the level of digital information utilization, as shown in Table 1, to prepare the dependent variable. All the questions had a 4-point scale (strongly disagree; disagree; agree; strongly agree). The average score of the 18 questions was calculated (average: 2.0000749), and those respondents whose score was lower than the average were classified as a group with a low digitalization level and assigned a value of zero (0) (1,193, 51.87%). Those respondents whose score was higher than the average were classified as a group with a high digitalization level and assigned a value of one (1) (1,107, 48.13%). Those respondents who were assigned a value of zero are affected by the digital divide. The dependent variable was named the ‘digitalization level,’ as used by the National Information Society Agency, to conceptualize the 18 questions. Fig 2 shows the ratio of the dependent variable.

### Independent variables

When all variables are applied to the prediction, it is difficult to interpret the prediction model. In social sciences, prediction is important, but the interpretation of the results is also important. Therefore, to increase the convenience of model analysis while using many independent variables, this study was performed by extracting the variables that are highly important in predicting the ‘digitalization level.’ To do so, the variables with a missing value and those related to personal identification information, such as ID, were removed from the 215 variables included in the raw data. Then, the feature importance XGBoost algorithm was applied to the 65 remaining variables to derive three importance indices: weight, cover and gain. Then, 10 variables with high values for all three indices were selected as the final independent variables to establish a prediction model. Descriptions of these indices are described below.

- Weight: Number of times that a variable was used for tree segmentation
- Cover: Number of data separated by a variable
- Gain: The average training loss reduction gained when using a feature for splitting

### Prediction model analysis

In this study, LR, SVM, KNN, DT, XGBoost, and CNN were used as classification algorithms. Since this study attempted to compare the accuracy of classifiers, the parameter values of each classifier were set and analyzed as default values for a fair comparison. In the CNN, the Conv1D layer of the Keras open source library was used. A total of three layers were formed; the filter had structures of 10, 20, and 10; and the kernel size was 1. The rectified linear unit (ReLU) was used as the active function of each layer, and the sigmoid function was used as the active function of the last output dense layer. All other parameters used default values. In this study, 70% of the raw data were used as training data, and 30% were used as the test data. After training the prediction model with the training dataset, the accuracy of the prediction model was analyzed using the test dataset. The prediction model accuracy indices used in this study were accuracy, precision, recall and the F1-score. The definitions and formulas of the individual indices are shown below.

- Accuracy: Accuracy is the most intuitive performance measure and is simply the ratio of correctly predicted observations to the total observations.

$$\left(Accuracy\right)=\frac{TP+TN}{TP+FN+FP+TN}$$ (5)

- Precision: Precision is the ratio of correctly predicted positive observations to the total predicted positive observations.

$$\left(Precision\right)=\frac{TP}{TP+FP}$$ (6)

- Recall: Recall is the ratio of correctly predicted positive observations to all observations in the actual “yes” class.

$$\left(Recall\right)=\frac{TP}{TP+FN}$$ (7)

- F1-score: F1 score is the weighted average of precision and recall.

$$\left(F1-score\right)=2\times \frac{1}{\frac{1}{Precision}+\frac{1}{Recall}}=2\times \frac{Precision\times Recall}{Precision+Recall}$$ (8)

## Result

### Independent variables importance analysis

**Fig 3:**
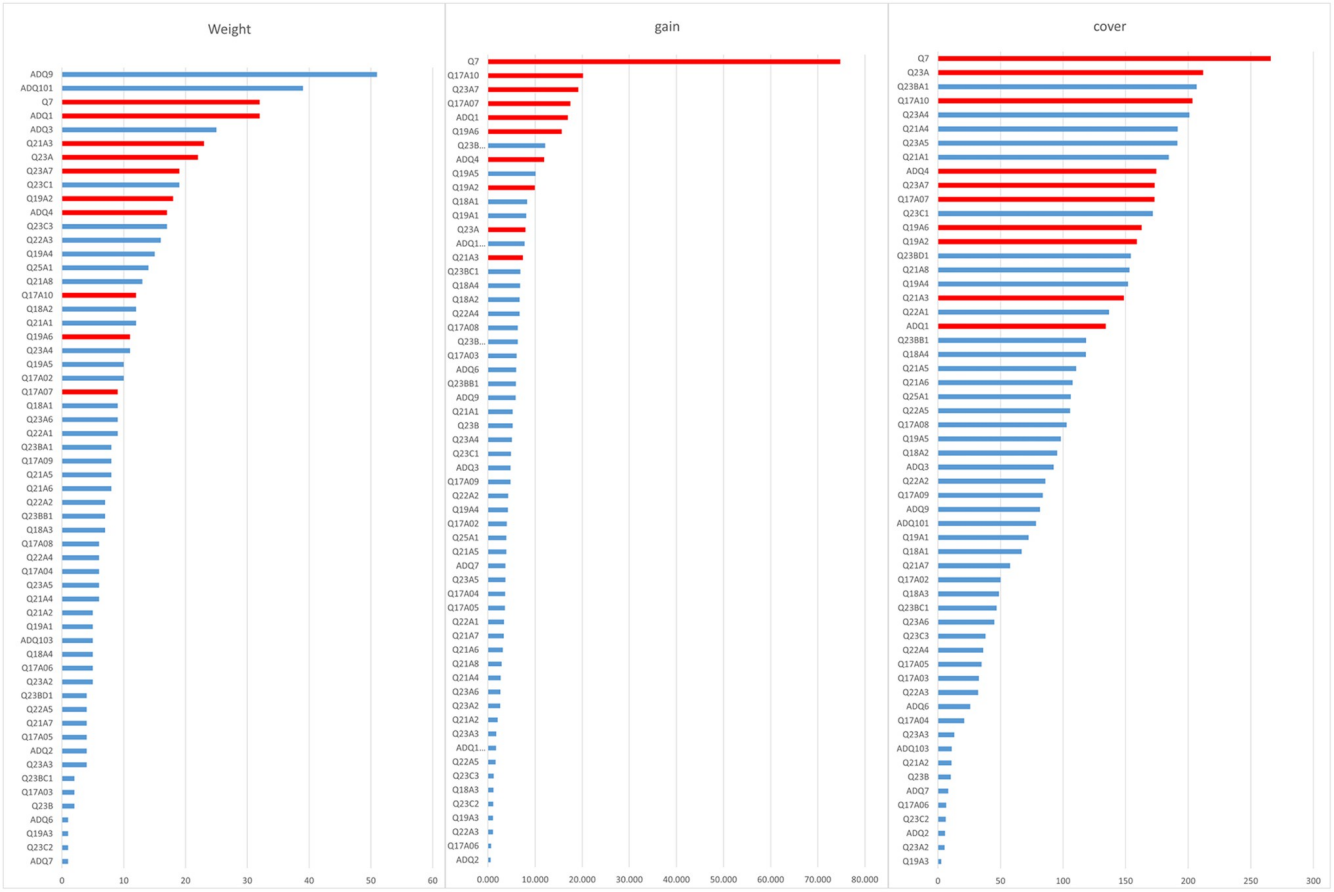
Importance of all independent variables.

The importance indices (weight, cover, and gain) of the independent variables were calculated by using the ‘plot_importance’ library of XGBoost to derive the variables with high importance in predicting the digital divide of elderly individuals. The independent variables are shown in Fig 3.

The values of the three importance indices differ between variables. Therefore, only one of the importance indices was selected and used in many previous studies, rather than handling the 3 importance indices at the same time. To overcome this limitation, the variables with high importance in terms of the 3 indices were extracted in this study by applying the ‘rank-based average calculation.’ For example, the 3 indices of the Q7 variable are weight: 3; cover: 1; and gain: 1. Therefore, the average is (3+1+1)/3 = 1.6. A lower average value means a higher rank. In this manner, the top-10-ranked variables were derived and used as the independent variables for predicting the digitalization level. The 10 variables are shown in Table 3.

**Table 3:** Independent variables.

Ranking	Variable Names	Independent Variables
1	Q7	Recent internet usage points
2	Q23A7	Digital consumption (purchasing daily necessities, food delivery, etc.) usage rate after COVID-19
3	Q17A10	Online and offline exchanges always allow you to meet new people.
4	ADQ1	Age
5	ADQ4	Final educational background
6	Q19A2	When using new technologies and products, I am confident that I will learn how to use them on my own.
7	Q23A	Changes in internet usage after COVID-19
8	Q19A6	I consider myself a lifelong learner and enjoy furthering my education as necessary
9	Q21A3	Satisfaction with social activities, such as community gatherings and community participation
10	Q17A07	While interacting with people online and offline, everyone in the world feels connected.

### Prediction model analysis results

The results of the analysis of the prediction model accuracy showed that among the 5 classifiers, the highest accuracy was achieved using the CNN-based model, followed by the XGBoost-based and LR-based models. The lowest accuracy was obtained using the DT-based model. The CNN is often used as a classifier for image type data. However, the results of this study showed that the CNN can be effectively used in classifying structured binary data, such as the data used in this study. Therefore, not only the traditional LR, SVM, KNN, DT and XGBoost methods but also the various artificial neural networks that are usually employed in images and videos can be applied to achieve interdisciplinary convergence and increase the accuracy of the machine learning-based prediction models. The analytical results are shown in Table 4.

**Table 4:** Prediction model analysis results.

Classification Model	Accuracy	Precision	Recall	F1-score
Decision Tree	0.726	0.715	0.729	0.722
Support Vector Machine	0.772	0.734	0.836	0.782
K-Nearest Neighbor	0.772	0.747	0.807	0.776
Logistic Regression	0.783	0.753	0.825	0.788
XGBoost	0.790	0.767	0.819	0.792
CNN	0.804	0.783	0.841	0.807

## Discussion

This study was conducted to predict the digital divide of older people in the current situation, where digital transformation is accelerated and noncontact environments are widespread. In particular, this study was conducted by using public data and machine learning, which have not been often used in previous studies on the digital divide, to establish digital divide prediction models and perform the relevant analyses. In this study, 10 variables with high importance in predicting the digital divide were derived, and they can be classified into **(1)** the demographic perspective (Variables 4 and 5), **(2)** the internet usage perspective (Variables 1, 2 and 7), **(3)** the self-efficacy perspective (Variables 6 and 8) and **(4)** the social connectedness perspective (Variables 3, 9 and 10). Why these 4 perspectives are important in predicting the digital divide of elderly individuals is discussed below.

First, the discussion of the digital divide basically focuses on demographic factors, such as age and education [8]. Those who are more advanced in age may have a low ICT utilization capability due to their decreased sensory functions and decreased cognitive abilities [23], and those who are more educated can easily acquire and utilize internet-based ICTs [73, 74]. Therefore, age and education have been argued to be the major factors in the digital divide, and this argument may have been reflected in the results of this study. Therefore, predicting the digital divide of older people requires careful consideration of demographic characteristics, such as age and education.

Second, the finding that a recent change in internet usage is important in predicting the digital divide of older people is related to the time when the survey that provided the data of this study was conducted. In 2020, when the survey was conducted, COVID-19 was spreading nationwide in Korea, thus inducing the application of a noncontact environment to many parts of daily living. Many activities, including business work, social activities and purchasing goods, have been carried out online; these activities can naturally increase internet usage. Those who have low internet usage despite their environment may be affected by a digital divide. Therefore, predicting the digital divide of older people requires careful consideration of the recent change in internet usage.

Third, the finding that confidence in learning and using ICTs is highly important in predicting the digital divide of elderly people suggests that confidence is the manifestation of self-efficacy. Self-efficacy is a user’s confidence that they can carry out a specific task or work by using the system [75, 76]. When people are confident that they have sufficient abilities to learn and use new skills, their resistance to acquiring these skills is decreased [77]. Therefore, self-efficacy is considered important in predicting the digital divide. In particular, according to innovation resistance theory [78], older people strongly resist acquiring and using new skills. However, high self-efficacy facilitates the adoption of new skills [79]. Therefore, predicting the digital divide of older people requires careful consideration of their attitude toward acquiring and using ICTs.

Fourth, the finding that the online social connectedness level and satisfaction are highly important in predicting the digital divide of older people suggests the manifestation of the characteristics of social connectedness. Elderly people tend to make social relationships and interact with others in an offline environment that they are familiar with rather than an online environment that they feel is difficult to access. However, older people usually cannot make new social relationships, as their social relationship formation is diminished due to their loss of social roles and physical and psychological limitations [80, 81]. Particularly in 2020, when the survey was conducted, social distancing was intensified in Korea to prevent COVID-19, thus making it more difficult to form offline-based social relationships with families and relatives. However, according to [55], online activities can help to form social relationships. The internet enables people to communicate with their children, friends and neighbors or make new friends who have common interests through online communities beyond the physical and social limitations of middle-aged or elderly people [82]. Moreover, online social connections allow for interactions with people who have totally different backgrounds regardless of political inclination, religion, sex and age [83]. Hence, the opportunities and activities that the internet environment provides to communicate with various kinds of people can not only enhance social connectedness but also naturally increase the ICT usage level, so such opportunities and activities can be considered factors that can reduce the digital divide. Therefore, predicting the digital divide of older people requires careful the consideration of their online social connectedness level and satisfaction with the connection.

Next, data from 2020 were studied. Since 2020, with the spread of COVID-19, social distancing intensified nationwide in Korea, and the majority of people were forced to use digital devices. Therefore, it was determined to be an important time to study the digital divide. In addition, data from 2019, before the outbreak of COVID-19, were collected and analyzed to compare the differences with the data from 2020. As a result of the analysis, the differences in predictors between 2019 and 2020 were determined to be the social support and social connection perspectives. In 2019, recognition of social support and desire for social connection was important, but in 2020, social support was not revealed as an important factor, and recognition of the degree of social connection was important. These results are recognized as the result of the strengthening of social distancing due to the increase in the nonface-to-face culture in 2020, making the level of actual connection through digital devices more important for survival than individual recognition that there are people who can provide assistance. This suggests that at a time when digital transformation is accelerating in various fields, support for strengthening practical connections through digital devices of the elderly is becoming more important than ever.

Then, the highest prediction accuracy of all studied classification algorithms was achieved using the CNN (accuracy: 80.4%). The CNN basically converts three-dimensional data into one-dimensional vectors and then calculates probability values, so it is recognized as effectively predicting one-dimensional data, as it does in this study. Meanwhile, the second highest accuracy (accuracy: 79%), was achieved using XGBoost, which performs well in international machine learning competitions such as Kaggle. Logistic regression analysis, which is considered a traditional method, ranked third, with good performance (accuracy: 78.3%). Smaller accuracies were obtained using SVM and KNN compared with the top three classification algorithms (CNN, XGBoost, LR), but there was not a significant difference. In contrast, the smallest value among all classification algorithms was observed when using DT. This result is recognized as a result of the characteristic that the decision tree is overfitted to the training data and is weak in predicting new data.

## Conclusion

The academic significance of this study is that the CNN, which is often employed in image and video processing, was extended and applied to a social science field using structured data to improve the accuracy of the prediction model. The Korean government announced the Digital New Deal Plan to overcome the economic crisis caused by COVID-19, and one of the major goals of the plan is to resolve the digital divide. The prediction model establishment method and the analytical method proposed in this article can be used by the relevant governmental authorities to classify elderly individuals who are affected by the digital divide. In addition, these methods can be used to predict the 40–49-year-olds who are likely to experience a digital divide as they age so that preemptive actions can be taken. The practical significance of this study is that, as a method for managing individuals who are affected by a digital divide, the self-efficacy perspective about acquiring and using ICTs and the socially connected perspective are suggested in addition to the demographic perspective and the internet usage perspective. This study is limited because it lacks comparisons with other generations because this study was conducted only with elderly individuals as the subjects and specifically focused on the digital divide of elderly individuals. Future studies need to be conducted in which older people are compared with other generations to investigate the patterns of the digital divide in different generations and extend the discussion on the digital divide.

## References

[1] Alhassan M.D., Adam I.O. 2021. The effects of digital inclusion and ICT access on the quality of life: A global perspective. Technology in Society. 64, 101511, doi: 10.1016/j.techsoc.2020.101511

[2] Jones P., Wynn M., Hillier D., Comfort D. 2017. The sustainable development goals and information and communication technologies, Indonesian Journal of Sustainability Accounting and Management, 1 (1), 1–15.

[3] Atkinson J., Black R., Curtis A. 2008. Exploring the digital divide in an Australian regional city: A case study of Albury. Australian Geographer, 39 (4), 479–493. doi: 10.1080/00049180802419203

[4] Graham R. 2010. Group differences in attitudes towards technology among Americans. New Media & Society, 12 (6), 985–1003. doi: 10.1177/1461444809341436

[5] Casado-Muñoz R., Lezcano F., Rodríguez-Conde M. J. 2015. Active ageing and access to technology: An evolving empirical study. Comunicar, 23 (45), 37–46.

[6] DiMaggio, P., Hargittai, E. 2001. From the ’digital divide’ to ’digital inequality’: Studying internet Use as Penetration increases. Working papers #15. Center for arts and cultural policy studies. Woodrow Wilson school. Princeton university.

[7] Selwyn N. 2004. Reconsidering political and popular understandings of the digital divide. New media & society. 6 (8), 341–362.

[8] Van Dijk J.A. 2006. Digital divide research, achievements and shortcomings. Poetics 34 (4), 221–235.

[9] Norris P. 2001. Digital divide? Civic engagement. Information poverty and the internet in democratic societies. New York: Cambridge university. Press.

[10] OECD. 2001. Understanding the digital divide.

[11] Van Dijk J.A. 2002. A framework for digital divide research. Electronic journal of communication. 12 (1&2), 1–7.

[12] Min Y. 2011. The digital divide among internet users: an analysis of digital access, literacy, and participation. Journal of Communication Research, 48 (1), 150–187.

[13] Hargittai E. 2010. Digital na(t)ives? Variation in internet skills and uses among members of the ‘net generation’. Sociological inquiry. 80 (1), 92–113.

[14] Pearce K.E., Rice R.E. 2017. Somewhat separate and unequal: Digital divides, social networking sites, and capital-enhancing activities. Social media + society. 2017 (6), 1–16.

[15] Hwang E.H., Shin S.J., Jung D.Y. 2011. A study of the pattern of elderly’s internet usage, self-efficacy, and self-esteem. Journal of Korean public health nursing. 25 (1), 118–128.

[16] Neves B.B., Amaro F., Fonseca J.R. 2013. Coming of (old) age in the digital age: ICT usage and non-usage among older adults. Sociological research online. 18 (2), 22–35.

[17] Haight M., Quan-Haase A., Corbett B.A. 2014. Revisiting the digital divide in Canada: The impact of demographic factors on access to the internet, level of online activity, and social networking site usage. Information communication & society. 17 (4), 503–519.

[18] Eastin M.S., Ciccirillo V., Mabry A. 2015. Extending the digital divide conversation: Examining the knowledge gap through media expectancies. Journal of broadcasting & electronic media. 59 (3), 416–437.

[19] Friemel T.N. 2016. The digital divide has grown old: Determinants of a digital divide among seniors. New media & society. 18 (2), 313–331.

[20] Puspitasari L., Ishii K. 2016. Digital divides and mobile internet in Indonesia: Impact of smartphones. Telematics and informatics. 33 (2), 472–483.

[21] Scheerder A., Van Deursen A., Van Dijk J. 2017. Determinants of Internet skills, uses and outcomes. A systematic review of the second- and third-level digital divide. Telematics and Informatics. 34 (8), 1607–1624.

[22] Statistics Korea. 2020. 2020 statistics on the elderly.

[23] Chen K., Chan A.H.S. 2011. A review of technology acceptance by older adults. Gerontechnology. 10 (1), 1–12.

[24] Kiel J.M. 2005. The digital divide: Internet and e-mail use by the elderly. Medical informatics and the internet in medicine. 30 (1), 19–23. doi: 10.1080/14639230500066900 16036627

[25] Lissitsa S., Chachashvili-Bolotin S. 2016. Life satisfaction in the internet age-changes in the past decade. Computers in human behavior. 54 (2016), 197–206.

[26] Khalaila R., Vitman-Schorr A. 2018. Internet use, social networks, loneliness, and quality of life among adults aged 50 and older: mediating and moderating effects. Quality of life research. 27 (2018), 479–489. doi: 10.1007/s11136-017-1749-4 29210015

[27] Berner J., Aartsen M., Deeg D. 2017. Predictors in starting and stopping Internet use between 2002 and 2012 by Dutch adults 65 years and older. Health informatics journal. 25 (3), 715–730. doi: 10.1177/1460458217720398 28747085 PMC6769282

[28] Eastman J.K., Iyer R. 2005. The impact of cognitive age on Internet use of the elderly: An introduction to the public policy implications. International journal of consumer studies. 29 (2), 125–136.

[29] Czaja S.J., Lee C.C. 2007. The impact of aging on access to technology. Universal access in the information society. 5 (4), 341.

[30] National Information Society Agency. 2020. 2020 The report on the digital divide.

[31] Korea Creative Content Agency. 2019. Content Industry 2018 Settlement and Forecast Report for 2019.

[32] Van Jaarsveld G.M. 2020. The effects of COVID-19 among the elderly population: A case for closing the digital divide. Frontiers in psychiatry. 11:577427. doi: 10.3389/fpsyt.2020.577427 33304283 PMC7693633

[33] Blank G., and Groselj D. 2014, Dimensions of Internet use: amount, variety, and types. Information, Communication & Society, 17 (4), 417–435.

[34] Hatlevik O.E., Guðmundsdóttir G.B., and Loi M. 2015, Examining Factors Predicting Students’ Digital Competence. Journal of Information Technology Education: Research, 14, 123–137.

[35] van Deursen A.J., and van Dijk J.A. 2010, Measuring internet skills. International Journal of Human-Computer Interaction, 26 (10), 891–916.

[36] van Deursen A.J., and van Dijk J.A. 2014, The digital divide shifts to differences in usage. New Media & Society, 16 (3), 507–526.

[37] Alqurashi E. 2018, Predicting student satisfaction and perceived learning within online learning environments. Distance Educ. 40 (1), 133–148, doi: 10.1080/01587919.2018.1553562

[38] Choubin B., Khalighi-Sigaroodi S., Malekian A., and Kişi O. 2016, Multiple linear regression, multi-layer perceptron network and adaptive neuro-fuzzy inference system for forecasting precipitation based on large-scale climate signals. Hydrological Sciences Journal, 61 (6), 1001–1009, doi: 10.1080/02626667.2014.966721

[39] Gaudart J., Giusiano B., and Huiart L. 2004, Comparison of the performance of multi-layer perceptron and linear regression for epidemiological data. Computational Statistics & Data Analysis, 44 (4), 547–570, doi: 10.1016/s0167-9473(02)00257-8

[40] Krzywinski M., and Altman N. 2015, Multiple linear regression. Nature Methods, 12 (12), 1103–1104, doi: 10.1038/nmeth.3665 26962577

[41] Hew K.F., Hu X., Qiao C., and Tang. Y. 2019, What predicts student satisfaction with MOOCs: A gradient boosting trees supervised machine learning and sentiment analysis approach. Computers & Education, 145, doi: 10.1016/j.compedu.2019.103724

[42] Akinwand M.O., Hussaini G.D., and Shehu U.G. 2015, Identifying the Limitation of Stepwise Selection for Variable Selection in Regression Analysis. American Journal of Theoretical and Applied Statistics, 4 (5), 414–419. doi: 10.11648/j.ajtas.20150405.22

[43] Smith G. 2018, Step away from stepwise. Journal of Big Data, 5 (32), doi: 10.1186/s40537-018-0143-6

[44] Ahmed N.K., Atiya A.F., Gayar N.E., and El-Shishiny H. 2010, An Empirical Comparison of Machine Learning Models for Time Series Forecasting. Econometric Reviews, 29 (5–6), 594–621, doi: 10.1080/07474938.2010.481556

[45] Balfer J., and Bajorath J. 2015, Systematic Artifacts in Support Vector Regression-Based Compound Potency Prediction Revealed by Statistical and Activity Landscape Analysis. PLoS ONE, 10 (3), e0119301. doi: 10.1371/journal.pone.0119301 25742011 PMC4350943

[46] Cutler, A., Cutler, D.R., and Stevens, J.R. 2012, Random Forests. Ensemble Machine Learning. 157–175.

[47] Jeong J.H., Resop J.P., Mueller N.D., Fleisher D.H., Yun K., and Butler E.E. 2016, Random Forests for Global and Regional Crop Yield Predictions. PLoS ONE, 11 (6), e0156571. doi: 10.1371/journal.pone.0156571 27257967 PMC4892571

[48] Maity R., Bhagwat P.P., and Bhatnagar A. 2010, Potential of support vector regression for prediction of monthly streamflow using endogenous property. Hydrological Process, 24 (7), 917–923. doi: 10.1002/hyp.7535

[49] Wang Y., and Wang T. 2020, Application of improved LightGBM model in blood glucose prediction. Applied Sciences, 10: 3227.

[50] Zhang J., Mucs D., Norinder U., and Svensson F. 2019, LightGBM: An Effective and Scalable Algorithm for Prediction of Chemical Toxicity–Application to the Tox21 and Mutagenicity Datasets, Journal of Chemical Information and Modeling, 4150–4158, doi: 10.1021/acs.jcim.9b00633 31560206

[51] Murphy, K.P. 2012, Machine learning: a probabilistic perspective. MIT press.

[52] Ngai E.W.T., Hu Y., Wong Y.H., Chen Y., and Sun X. 2011, The application of data mining techniques in financial fraud detection: A classification framework and an academic review of literature. Decision Support Systems, 50 (3), 559–569, doi: 10.1016/j.dss.2010.08.006

[53] Woo, J.P. 2022. Concepts and Understanding of Structural Equations Model. Hannarae academy.

[54] Varian H.R. 2014. Big data: New tricks for econometrics. Journal of economic perspectives. 28 (2), 3–28.

[55] Lee B., Kim. Y. 2010. An empirical study of the effectiveness of internet-using of the elderly people: Focusing on social network and human reliances. Korean policy sciences review. 14 (3), 79–105.

[56] Burgers C.J.C. 1998. A tutorial on support vector machines for pattern recognition. Data mining and knowledge discovery. 2, 121–167.

[57] Chen, T., Guestrin, C. 2016. XGBoost: A scalable tree boosting system. In proceedings of the 22Nd ACM SIGKDD international conference on knowledge discovery and data mining. 785–794.

[58] Zhao, K., Wang, C. 2017. Sales forecast in e-commerce using convolutional neural network. https://arxiv.org/abs/1708.07946.

[59] Wang, H., Wang, J., Cao, L., Li, Y., Sun, Q., Wang, J. 2021. A stock closing price prediction model based on CNN-BiSLSTM. Complexity. 2021, Article ID 5360828.

[60] Jia Y., Kaul C., Lawton T., Murray-Smith R., Habli I. 2021. Prediction of weaning from mechanical ventilation using Convolutional Neural Networks. Artificial intelligence in medicine. 117 (2021), 102087. doi: 10.1016/j.artmed.2021.102087 34127233

[61] Awoyemi, J.O., Adetunmbi, A.O., and Oluwadare, S.A. 2017, Credit card fraud detection using machine learning techniques: A comparative analysis. 2017 International Conference on Computing Networking and Informatics (ICCNI), 1–9.

[62] Jung J.S., Park S.J., Kim E.Y., Na K.S., Kim Y.J., and Kim K.G. 2019, Prediction models for high risk of suicide in Korean adolescents using machine learning techniques. PLoS ONE, E 14(6): e0217639, doi: 10.1371/journal.pone.0217639 31170212 PMC6553749

[63] Talpur B.A., and O’sullivan D. 2020, Cyberbullying severity detection: A machine learning approach. PLoS ONE, 15 (10) e0240924, doi: 10.1371/journal.pone.0240924 33108392 PMC7591033

[64] Ho I.M.K., Cheong K.Y., and Weldon A. 2021, Predicting student satisfaction of emergency remote learning in higher education during COVID19 using machine learning techniques. PLoS ONE, 16 (4), e0249423, doi: 10.1371/journal.pone.0249423 33798204 PMC8018673

[65] Abu Saa A., Al-Emran M., and Shaalan K. 2019, Factors Affecting Students’ Performance in Higher Education: A Systematic Review of Predictive Data Mining Techniques. Technology, Knowledge and Learning, 24, 567–598, doi: 10.1007/s10758-019-09408-7

[66] Kovačić, Z.J., Vukmirović, D. 2008. ICT adoption and the digital divide in Serbia: factors and policy implications. Proceedings of the informing science & it education conference. (Insite).

[67] Coria S.R., Mondragón-Becerra R., Pérez-Meza M., Ramírez-Vásquez S.K., Martínez-Peláez R., Barragán-López D., et al. 2013. CT4RDD: Classification trees for research on digital divide. Expert systems with applications. 40 (14), 5779–5786.

[68] Hidalgo A., Gabaly S., Morales-Alonso G. 2020. The digital divide in light of sustainable development: An approach through advanced machine learning techniques. Technological forecasting and social change. 150 (2020), 119754.

[69] Oyeyemi G.M., Ogunjobi E.O., and Folorunsho A.I. 2015, On Performance of Shrinkage Methods–A Monte Carlo Study. International Journal of Statistics and Applications, 5 (2), 72–76.

[70] Alizamir M., Kisi O., Ahmed A.N., Mert C., Fai C.M., and Kim S. 2020, Advanced machine learning model for better prediction accuracy of soil temperature at different depths. PLoS ONE, 15 (4), e0231055, doi: 10.1371/journal.pone.0231055 32287272 PMC7156082

[71] Povak N.A., Hessburg P.F., McDonnell T.C., Reynolds K.M., Sullivan T.J., and Salter R.B. 2014, Machine learning and linear regression models to predict catchment-level base cation weathering rates across the southern Appalachian Mountain region, USA. Water Resource Research, 50 (4), 2798–2814, doi: 10.1002/2013wr014203

[72] Rai A. 2020, Explainable AI: from black box to glass box. Journal of the Academy of Marketing Science, 48, 137–141. doi: 10.1007/s11747-019-00710-5

[73] Mollenkopf H., Kaspar R. 2005. Ageing in rural areas of east and west Germany: Increasing similarities and remaining differences. European journal of ageing. 2 (2), 120–130. doi: 10.1007/s10433-005-0029-2 28794724 PMC5547681

[74] Korupp S.E. 2006. No man is an island: The influence of knowledge, household settings, and social context on private computer use. International journal of internet science. 1 (1), 45–57.

[75] Hsu M.H., Chiu C.M. 2004. Internet self-efficacy and electronic service acceptance. Decision support systems. 38 (3), 369–381.

[76] Isaac V., Pit S.W., Mclachlan C.S. 2018. Self-efficacy reduces the impact of social isolation on medical student’s rural career intent. BMC medical education. 18 (42), doi: 10.1186/s12909-018-1142-1 29554908 PMC5859449

[77] Basak E.B., Gumussoy C.A., Calisir F. 2015. Examining the factors affecting PDA acceptance among physicians: An extended technology acceptance model. Journal of healthcare engineering. 16 (3), 399–418. doi: 10.1260/2040-2295.6.3.399 26753441

[78] Ram S. 1987. A model of innovation resistance. Advances in consumer research, 14 (1), 208–212.

[79] Bandura A. 1988. Self-efficacy conception of anxiety. Anxiety research. 1 (1988), 77–98.

[80] Chung S., Sung M. 2012. Relationship between social capital and life satisfaction: A comparison of three difference age groups. Health and social welfare review. 32 (4), 249–272.

[81] Kim K., Lee S., Yoon H., Kwon G. 2015. The effects of social capital of old-old elderly of more than 70-year-old on their health-related quality of life. Journal of the Korea academia-industrial cooperation society. 16 (6), 3889–3901.

[82] Jun D. 2015. Effects of the Elderly computer/internet competence on life satisfaction. Korean journal of local government & administration studies. 29 (3), 389–409.

[83] Williams D. 2006. On and off the’net: Scales for social capital in an online era. Journal of computer-mediated communication. 11 (2), 593–628.

